# Aortic and Carotid Arterial Stiffness and Epigenetic Regulator Gene Expression Changes Precede Blood Pressure Rise in Stroke-Prone Dahl Salt-Sensitive Hypertensive Rats

**DOI:** 10.1371/journal.pone.0107888

**Published:** 2014-09-17

**Authors:** Victoria L. Herrera, Julius L. Decano, Nicholas Giordano, Ann Marie Moran, Nelson Ruiz-Opazo

**Affiliations:** Whitaker Cardiovascular Institute, Department of Medicine, Boston University School of Medicine, Boston, Massachusetts, United States of America; The University of Manchester, United Kingdom

## Abstract

Multiple clinical studies show that arterial stiffness, measured as pulse wave velocity (PWV), precedes hypertension and is an independent predictor of hypertension end organ diseases including stroke, cardiovascular disease and chronic kidney disease. Risk factor studies for arterial stiffness implicate age, hypertension and sodium. However, causal mechanisms linking risk factor to arterial stiffness remain to be elucidated. Here, we studied the causal relationship of arterial stiffness and hypertension in the Na-induced, stroke-prone Dahl salt-sensitive (S) hypertensive rat model, and analyzed putative molecular mechanisms. Stroke-prone and non-stroke-prone male and female rats were studied at 3- and 6-weeks of age for arterial stiffness (PWV, strain), blood pressure, vessel wall histology, and gene expression changes. Studies showed that increased left carotid and aortic arterial stiffness preceded hypertension, pulse pressure widening, and structural wall changes at the 6-week time-point. Instead, differential gene induction was detected implicating molecular-functional changes in extracellular matrix (ECM) structural constituents, modifiers, cell adhesion, and matricellular proteins, as well as in endothelial function, apoptosis balance, and epigenetic regulators. Immunostaining testing histone modifiers Ep300, HDAC3, and PRMT5 levels confirmed carotid artery-upregulation in all three layers: endothelial, smooth muscle and adventitial cells. Our study recapitulates observations in humans that given salt-sensitivity, increased Na-intake induced arterial stiffness before hypertension, increased pulse pressure, and structural vessel wall changes. Differential gene expression changes associated with arterial stiffness suggest a molecular mechanism linking sodium to full-vessel wall response affecting gene-networks involved in vascular ECM structure-function, apoptosis balance, and epigenetic regulation.

## Introduction

Multiple studies of arterial stiffness in humans, typically measured as carotid-femoral pulse wave velocity (cfPWV) and hence referred to as aortic stiffness, have demonstrated that arterial stiffness is an independent risk factor for cardiovascular outcomes in hypertensive patients including mortality [1], myocardial infarction [2], fatal stroke in essential hypertension [3], and cerebral microbleeds which predict cerebral hemorrhage [4]. In fact, the association between aortic stiffness and coronary heart disease, as well as stroke, remains after adjusting for age, sex, blood pressure, BMI and other known predictors of cardiovascular disease including the Framingham risk factors, thus suggesting that arterial stiffness (measured cfPWV) is a better predictor than each of these known risk factors for cardiovascular disease outcomes [5]. These observations have been corroborated in larger and more recent studies reporting that arterial stiffness is associated or predicts a) coronary heart disease, stroke and cardiovascular disease events independent of and better than conventional risk factors [6], b) coronary artery disease in patients with stroke/transient ischemic attacks (after adjustment for the Framingham Risk Score) [7], and c) all-cause mortality and cardiovascular events in chronic kidney disease [8].

Addressing whether arterial stiffness precedes vascular diseases or is secondary to it, association studies in humans reported that arterial stiffness was an independent predictor of progression to hypertension in non-hypertensive subjects [9], and that increased aortic stiffness, decreased aortic strain and decreased aortic distensibility have been observed in both hypertensive patients and young patients with prehypertension [10]. Concordantly, a 2-time point prospective study in the Framingham Offspring cohort observed that aortic stiffness, brachial pulse pressure, peripheral wave reflection, and central pressure pulsatility; along with macro- and micro-vascular endothelial function jointly preceded essential hypertension [11]. Altogether, these multiple studies showed that arterial stiffness preceded hypertension and predicts its target organ complications independent of other risk factors studied (diabetes, smoking, cholesterol levels, and waist circumference). Although these studies did not report concurrent analysis of sodium intake as risk factor, other studies in different populations, China [12], Australia [13] and Europe [14], have shown association between dietary sodium and arterial stiffness independent of blood pressure and measurement modalities [15]–[17]. These observations suggest that, just as sodium is correlated with arterial stiffness and arterial stiffness precedes hypertension, sodium-induced arterial stiffness presents as a pathogenic paradigm for salt-sensitive hypertension and its target organ complications. This paradigm is concordant with aging-associated arterial stiffness [18], aging-associated salt-sensitivity [19], [20] and the observation of increased hypertension with aging [21], [22].

Because of the consistent predictive association of arterial stiffness and hypertension end-organ diseases, arterial stiffness is posited as a robust therapeutic target to decrease hypertension target organ complications [23] which, although reduced in prevalence do still persist with current anti-hypertensive medications keeping cardiovascular disease and stroke as the top three causes of mortality. Notably, some anti-hypertension therapies have been found to reduce arterial stiffness such as inhibitors of the renin-angiotensin aldosterone system and diuretics but not beta-blockers [24], but more studies, if not new drugs, are needed given that overall, the prevalence of stroke, heart disease, and chronic kidney disease persist higher than expected from the level of reduction in hypertension attained. Given these observations in clinical studies, further analysis of arterial stiffness is imperative and mandates systematic dissection of causal mechanisms of arterial stiffness in a modeled biological context that recapitulates the pathogenic triad of arterial stiffness, hypertension and end-organ complications.

Altogether, these association and treatment response studies make the study of mechanisms underlying the predictive value of arterial stiffness more complex but imperative. Current biomechanical hypotheses implicate arterial stiffness and pressure pulsatility as directly altering the structure and function of small arteries [25], concordant with deductions from animal model studies that arterial stiffness and increased pulse pressure stimulate vessel wall hypertrophy and remodeling, as well as rarefaction in the microcirculation [26], but which require further study on the interactions of arterial stiffness and final end-organ damage [27]. Current biostructural insight implicate increased aortic collagen, hyaluronan content, and/or increased attachments between vascular smooth muscle cells and collagen through increased fibronectin and its receptor, α5β integrin receptor [28]. These however do not link risk factors of arterial stiffness to arterial stiffness.

Causal factors that contribute to arterial stiffness – prior to hypertension, and prior to end-organ complications – require elucidation since majority of studies found no independent association between cfPWV and sex, total cholesterol, LDL, HDL, triglycerides, smoking, diabetes, waist circumference or BMI [29], [30]. However, the relationship of sodium intake and arterial stiffness was not included in these studies. Intriguingly, in vitro experiments showed that sodium overload decreased the endothelial glycocalyx sodium barrier by ∼50% increasing endothelial stiffness by 130% [31], stiffens endothelial cell actin web [32], and that “mechanical stiffness determines nitric oxide (NO) release and not vice versa” [33] with EC stiffness and NO release inversely related [34], and that sodium downregulates eNOS expression [35] and increases intracellular production of competitive eNOS inhibitor asymmetrical dimethyl-L-arginine [36]. Additionally, increased sodium content in the interstitium has been implicated to contribute to arterial stiffness [37]. Altogether, these observations implicate sodium in arterial stiffness but that questions remain on mechanisms of sodium and arterial stiffness.

In order to address this gap in the elucidation of mechanisms of Na-induced arterial stiffness, studies need to be done in animal models using PWV as the measure. As a direct measure of arterial stiffness, Pulse Wave Velocity (PWV) measurement is generally accepted as the most straightforward, non-invasive, robust and reproducible method to determine arterial stiffness [38], and considered as the non-invasive ‘gold standard’ [39]. This is supported by epidemiological evidence [5], genetic studies of human aortic stiffness [25], [40], and in studies of aortic gene expression changes associated with human aortic stiffness [41] and of heritability in young adults [42]. With the availability of high-resolution ultrasound microimaging for rodents with 30–55 micron axial resolution, longitudinal, non-invasive measurement of PWV and strain are feasible. This has been validated in mouse common carotid artery [43].

In this study, we investigated the causal role of sodium in arterial stiffness and the temporal relationship of arterial stiffness and hypertension using high-resolution ultrasound microimaging and blood pressure telemetric measurements in a validated early-life Na-exposure-induced stroke-prone Dahl salt-sensitive (S) rat model with a cerebral microbleed phenotype [44]. In parallel, we also studied the potential structural and molecular changes associated with PWV changes in order to gain insight into the pathogenic significance of the physiologic measure of PWV, prior to hypertension in the juvenile stage, thus eliminating both aging and hypertension as co-factors.

## Results

In order to study age, hypertension, and sodium in one experimental system where these factors can be controlled, we tested whether sodium intake could increase arterial stiffness in a stroke-prone Dahl S model in the juvenile stage, at 3-weeks and 6-weeks of age, thus eliminating the two main factors associated with arterial stiffness – age and hypertension [29]. To gain insight into causal mechanisms of arterial stiffness, we evaluated the structural and molecular changes in the blood vessels exhibiting increased stiffness measured as PWV, and studied both the carotid artery and aorta.

### Arterial Stiffness Develops Prior to the Onset of Hypertension

**Figure 1:**
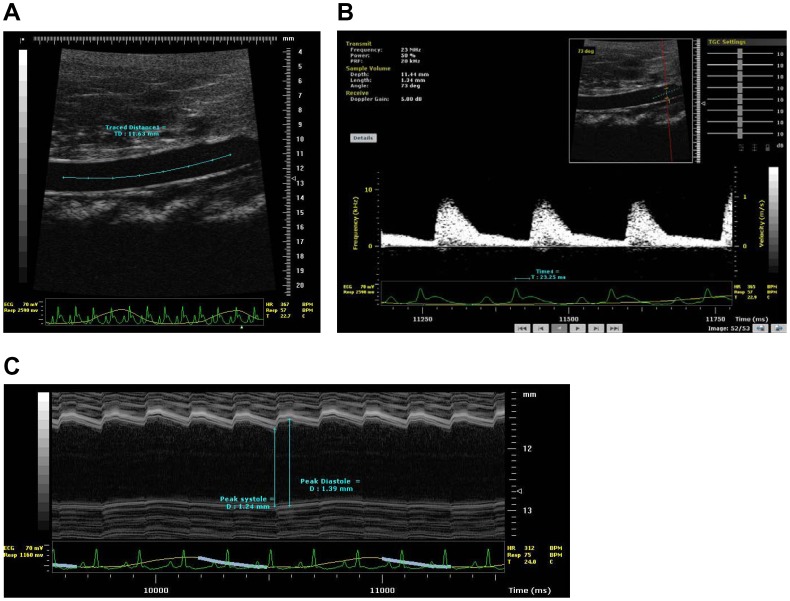
Representative images for pulse wave velocity (PWV) and strain measurements. **A**, Measurement of distance between two anatomical points along the abdominal aorta: proximal point after superior mesenteric artery branchpoint; distal point at site of crossing of renal vein. **B**, Representative Doppler frequency at distal point site: where renal vein crosses aorta. Integrated software for cursor-based measurement of distance given in mm (in **A**) and time in milliseconds from the peak of the ECG-R wave to the foot of the velocity upstroke (in **B**). **C**, Representative M-mode image for strain measurement in left carotid artery.

In order to model the association of salt-sensitivity, hypertension, and stroke, we studied the stroke prone Dahl salt-sensitive (S) rat model wherein stroke susceptibility is increased by developmental programming with increased early-life sodium exposure [44]. In this model, pups exposed to 0.4% NaCl during gestation exhibit increased susceptibility to stroke in Dahl-S rats (stroke-prone Dahl S rats, or SP), compared to pups exposed to 0.23% NaCl during gestation which are non-stroke prone (nSP) [44]. In order to study the impact of sodium alone on arterial stiffness, we studied arterial stiffness at two time points: 3- and 6-weeks of age, in order to eliminate age and hypertension. In order to make translatable deductions, we studied the non-invasive gold standard for arterial stiffness, pulse wave velocity (PWV) in two large arteries: the carotid artery and aorta. We measured PWV and arterial strain at two points along the common carotid artery, and in the abdominal aorta between the superior mesenteric artery and left renal artery as these measures gave more consistent measurements than carotid-femoral artery PWV as done in humans (Figure 1). Carotid artery PWV was previously validated [43].

**Figure 2:**
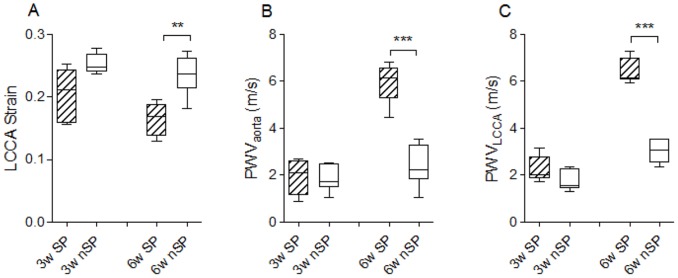
Arterial stiffness in stoke-prone (SP) and non stroke-prone (nSP) Dahl S female rats using pulse wave velocity (PWV) and arterial strain measured at three weeks and six weeks of age. Left common carotid arterial strain (**A**), aortic PWV (**B**) and left common carotid arterial PWV (**C**) were measured in SP and nSP Dahl S female rats at three weeks (3w) and six weeks (6w) of age. SP Dahl S females (3 weeks of age), n = 5; nSP Dahl S females (3 weeks of age), n = 6; SP Dahl S females (6 weeks of age), n = 5; nSP Dahl S females (6 weeks of age), n = 6. Values are presented as box-and-whisker plots with the ends of the whiskers representing the minimum and maximum of all of the data. **P<0.01, ***P<0.001 (One Way ANOVA followed by Holm-Sidak Test for multiple comparisons).

As shown in Figure 2, measurements of LCCA strain in female subjects at three weeks of age are equivalent between SP and nSP rats (Figure 2A). Likewise, in female rats at 3 weeks of age both SP and nSP rats demonstrate similar levels of PWV in aorta (Figure 2B) and left common carotid artery (Figure 2C). In contrast, arterial stiffness measurements at six weeks of age revealed a significant increase in arterial stiffness in SP rats compared with nSP rats (Figure 2). PWV values were significantly higher in SP female rats compared with nSP female rats in aorta (SP rats: 5.97±0.39, nSP rats: 2.39±0.36; P<0.001, Figure 2B) and LCCA (SP rats: 6.42±0.22, nSP rats: 3.02±0.20; P<0.001, Figure 2C). Measurement of vessel dimensions on histological preparations revealed equivalent values in vessel diameter and wall thickness between nSP and SP subjects in both aorta (aorta diameter nSP: 689.5±126.6 µm, SP: 680.6±53.2 µm; aorta wall thickness nSP: 75.28±2.6 µm, SP: 80.63±0.3 µm) and LCCA (LCCA diameter nSP: 608.2±46.4 µm, SP: 555.7±38.0 µm; LCCA wall thickness nSP: 48.78±4.0 µm, SP: 51.48±6.0 µm), thus affirming that the observed differential PWV values reflect differences in arterial stiffness. Concordantly, additional measurements of LCCA strain showed significantly decreased strain or distensibility in SP female rats (SP rats: 0.165±0.010, nSP rats: 0.235±0.013; P<0.01, Figure 2A) when compared with nSP female subjects at six weeks of age.

**Figure 3:**
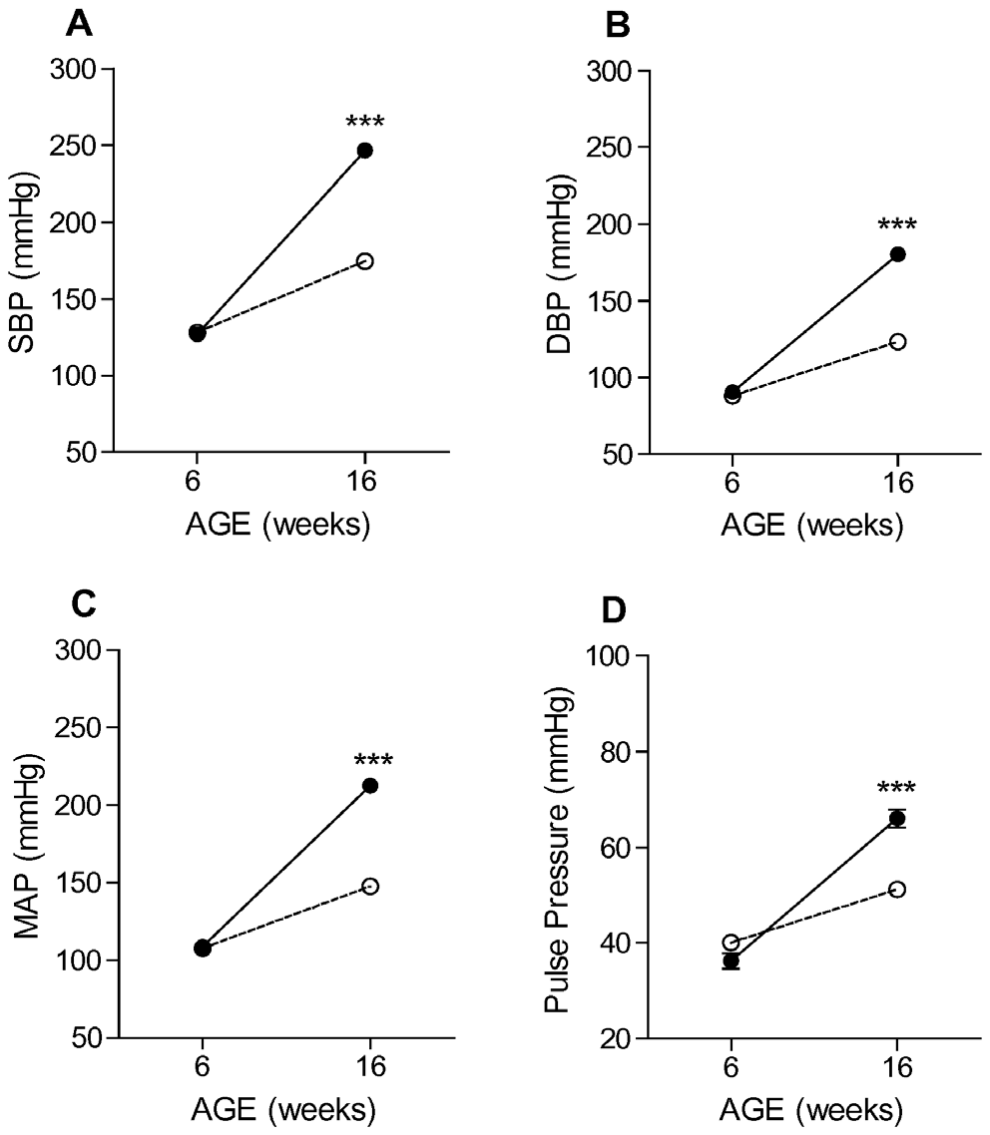
Development of high blood pressure in stoke-prone (SP) and non stroke-prone (nSP) Dahl S female rats. Systolic (**A**), diastolic (**B**), mean arterial (**C**) and pulse (**D**) pressures in SP Dahl S (n = 4, closed circles) and nSP Dahl S (n = 6, open circles) female rats. Blood pressure parameters were collected at 6 and 16 weeks of age. Values are means ± s.e.m. ***P<0.001, (Two Way ANOVA followed by Holm-Sidak Test for multiple comparisons).

To investigate potential differences in the development of high blood pressure in SP and nSP subjects, we measured blood pressure longitudinally by radiotelemetry in SP and nSP female rats at six weeks and sixteen weeks of age (Figure 3). At six weeks of age both SP and nSP rats exhibited comparable systolic (SP rats: 126.9±2.6, nSP rats: 128.1±2.1, Figure 3A), diastolic (SP rats: 90.6±1.6, nSP rats: 88.0±1.3, Figure 3B), mean arterial (SP rats: 108.7±2.0, nSP rats: 107.7±1.7, Figure 3C), and pulse (SP rats: 36.3±1.6, nSP rats: 40.1±1.3, Figure 3D) pressures, despite differences in arterial stiffness present at this age. This finding demonstrates that arterial stiffness precedes changes in blood pressure. As expected, at 16 weeks of age we detected significant differences in systolic (SP rats: 246.5±3.0, nSP rats: 174.5±2.1; P<0.001, Figure 3A), diastolic (SP rats: 180.5±1.9, nSP rats: 123.4±1.3; P<0.001, Figure 3B), mean arterial (SP rats: 212.6±2.3, nSP rats: 147.7±1.7; P<0.001, Figure 3C), and pulse (SP rats: 66.0±1.9, nSP rats: 51.2±1.3; P<0.001, Figure 3D) pressures between SP and nSP subjects indicating that increases in arterial stiffness precedes the increase in all measures of blood pressure.

### Absence of Structural Changes in LCCA and Aortic Vessels in SP Dahl S Rats at Six Weeks of Age

To determine whether micro-structural changes are present at 6 weeks of age when PWV changes occur but prior to BP elevation, we analyzed Masson Trichrome and H&E stained serial sections of aortic and left common carotid artery (LCCA)-sections from SP and nSP rat arteries respectively at 6-weeks of age. These comprised of sections obtained from the proximal and distal ends of arterial segments used for analysis of PWV and strain, with the middle segments for pathway-specific molecular analysis presented below. This segment-specific analysis validates the analysis of structural changes associated with arterial stiffness measures, PWV and strain.

**Figure 4:**
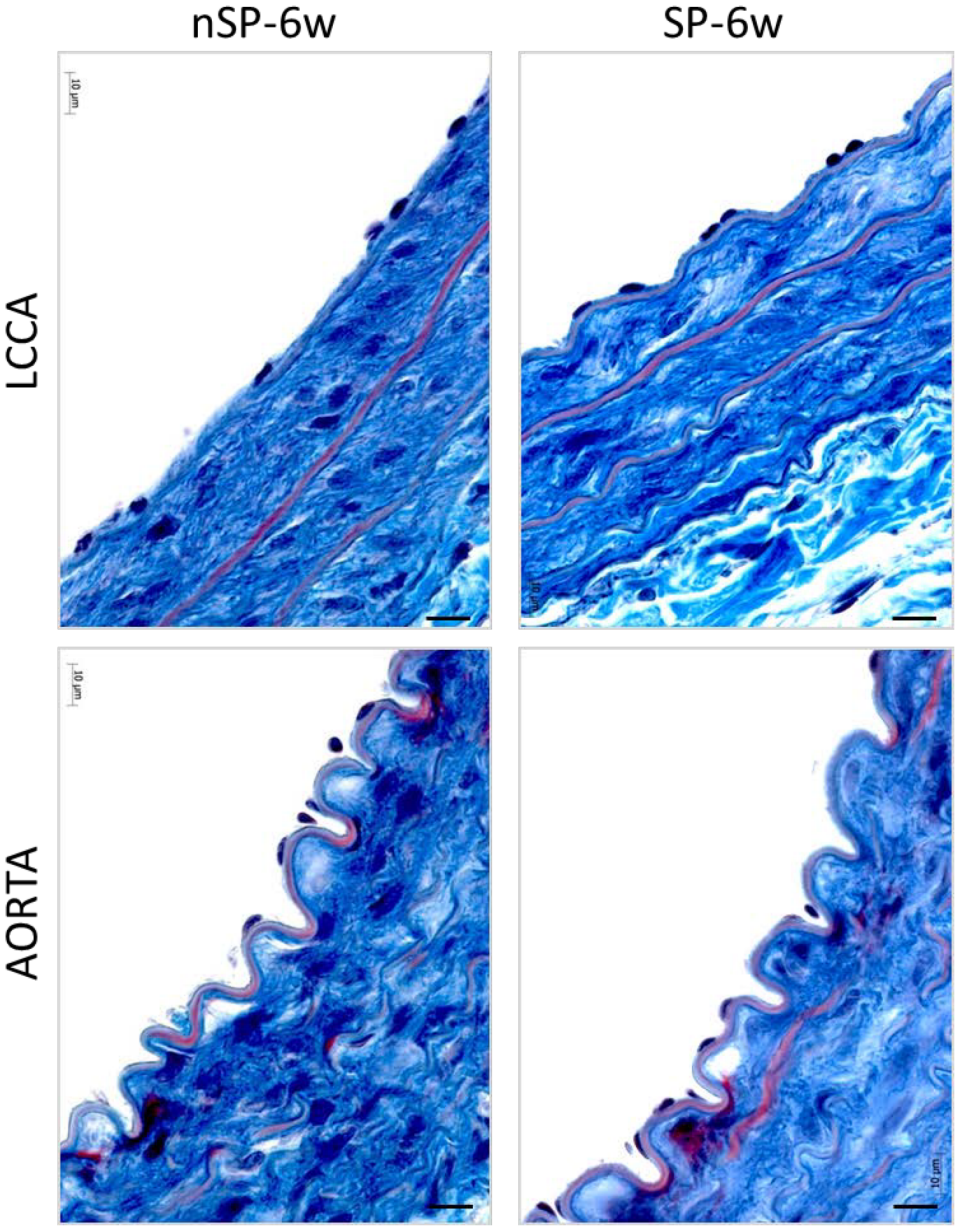
Representative histological micrographs of aortic and left common carotid artery (LCCA) sections from stoke-prone (SP) and non stroke-prone (nSP) Dahl S female rats at six weeks of age. Masson-trichrome stained sections of LCCA and abdominal aorta taken at the site of PWV measurement. Bar = 10 microns.

As shown in a representative section in Figure 4, no structural changes were observed in both LCCA and aortic Masson-trichrome stained serial sections. The endothelia were equivalently intact with minor thickening in some spots; there was no neointimal hyperplasia, the elastic laminae were intact and parallel, and the collagen content in the media and adventitia were relatively unchanged on Masson-Trichrome staining (Figure 4). These observations eliminate classical structural alterations associated with arterial stiffness such as vessel wall hypertrophy or remodeling [45].

### Vessel-specific differential gene expression changes in 6w-old SP Dahl-S rats

**Figure 5:**
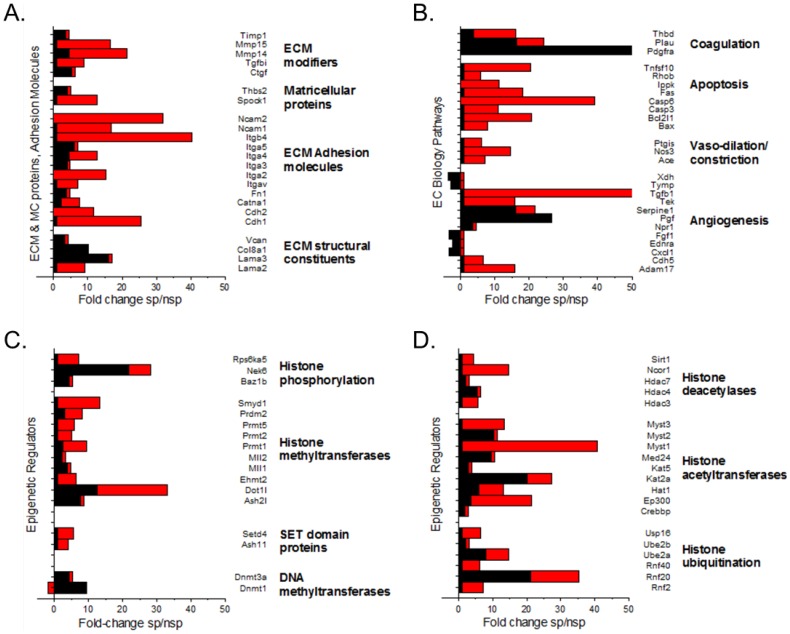
RT-PCR array profiling in aortas and left common carotid artery (LCCA) of stoke-prone (SP) and non stroke-prone (nSP) Dahl S female rats at six weeks of age. Pathway-specific RT2-qPCR array comparative analysis of gene expression changes in LCCA (red bars) and aorta (black bars) at 6-weeks of age representing ratio of SP/nSP RNA levels. A) Extracellular matrix (ECM) and matricellular (MC) protein pathway-specific significant gene changes. B) Endothelial Cell (EC) Biology pathway-specific gene changes. C and D) Epigenetic regulator pathway-specific gene changes. SP (0.4% NaCl); nSP (0.23% NaCl) from gestation. Gene expression changes shown are limited to ≥ 2-fold change and p<0.01 in either vessel. Only statistically significant differences are presented (P<0.05, Two Way ANOVA followed by Holm-Sidak Test for multiple comparisons; Tables S1–S6).

To further investigate putative mechanisms that could underlie the functional differences in arterial stiffness detected between SP and nSP rats at six weeks of age, we performed pre-validated, pathway-specific reverse transcriptase, quantitative PCR (RT-qPCR) array analyses profiling the expression of 252 genes related to ECM homeostasis and endothelial cell function on steady-state total RNA samples isolated from the aortic and LCCA segments studies by PWV from SP and nSP rats at 3 weeks and six weeks of age (Figure 5 and Tables S1–S9). With limited sample amounts, and since RT-qPCR is used to confirm array chip-analyses in a 2-step process, the direct analysis by RT-qPCR using pre-validated pathway-specific arrays will provide robust quantitative analyses of genes interrogated in a one-step process, albeit querying less number of genes.

Overall, the analysis of gene expression changes at 6-weeks of age referenced to 3-week old artery segments detected more gene changes in the LCCA compared to the aorta in the three representative pathways interrogated: ECM homeostasis, EC biology, and epigenetic regulators represented by histone modification enzymes (Figure 5). While there were no evident structural changes detected at 1000-X oil immersion magnification on Masson-Trichrome stained sections, robust gene expression changes were detected in ECM modifiers, ECM adhesion molecules, ECM structural constituents, and matricellular proteins. Notably, changes in ECM structural components were greater in the aorta, while gene expression changes in ECM modifiers and adhesion molecules were detected to be greater in LCCA (Figure 5, Tables S1 and S2). Interestingly, increased collagen and integrins were detected in LCCA and aorta at 6-weeks, similar to prior reports [45], but differed in collagen and integrin isoforms. Similarly, analysis of genes involved in EC biology also detected gene expression changes in both LCCA and aorta, but with different gene profiles induced (Figure 5, Tables S3 and S4), thus indicating that vessel-specific molecular alterations are associated with Na-induced arterial stiffness. Notably, a complex molecular response is evident: apoptosis gene network balance was perturbed in LCCA towards apoptosis but not in the aorta; angiogenic genes were changed in both aorta and LCCA but differentially, while Ace, NOS3, TGFb1 are upregulated in LCCA but not in aorta, while PDGF-RA, FGF, and endothelin receptor type-A are markedly increased in aorta (Figure 5, Tables S3 and S4).

In order to test the hypothesis that gene expression changes in epigenetic regulators could provide a mechanism for the pathogenic continuum spanning arterial stiffness which precedes hypertension which precedes brain microvascular paucity which precedes stroke in this model [44], we analyzed changes in epigenetic regulators spanning histone activators, deactivators and modifiers, DNA methyltransferases and demethyltransferases (Figure 5-C,D, Tables S5 and S6). We focused on the gene expression changes of the epigenetic regulators per se rather than epigenetic marks on DNA because of the dynamic time-course of the latter and limits of samples for both RNA and DNA analysis in vessel segments studied by arterial stiffness PWV measures. Being all on the same epigenetic regulator array provides robust comparative analysis of different epigenetic regulators since genes interrogated on the array are subjected to identical conditions. As shown in Figure 5 (Tables S5 and S6), differential gene expression changes occur among epigenetic regulators of histones and DNA methylation states, with some identical changes in aorta and LCCA, but most differentially upregulated in either LCCA or aorta. Concordant with changes in ECM and EC steady states, overall gene expression changes among epigenetic regulators are greater in LCCA compared to aorta when using the pathway-specific array tested here. We note that gene changes detected are robust and specific given that there are more genes that are expressed but unchanged (Tables S7–S9).

### Immunohistofluorescence analysis detects epigenetic regulator changes in the endothelium, media and adventitia

**Figure 6:**
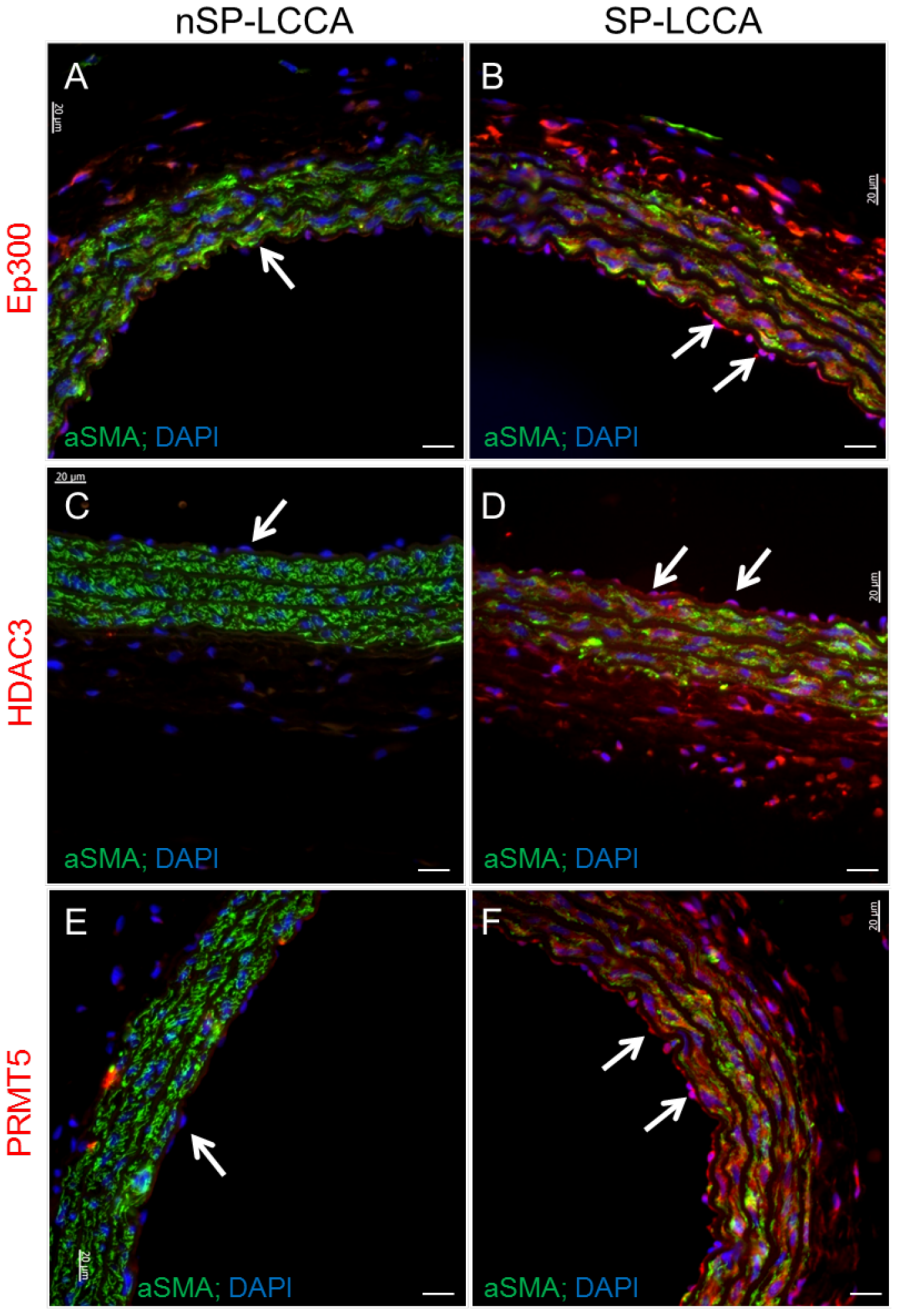
Representative immunofluorescence micrographs of left common carotid artery (LCCA) sections from stoke-prone (SP) and non stroke-prone (nSP) Dahl S female rats at six weeks of age. Immunohistofluorescence analysis of expression of epigenetic regulator genes detected to be significantly increased by pathway-specific RT2-qPCR array analysis in SP-LCCA compared to nSP-LCCA. Ep300, E1A binding protein p300 (histone acetyltransferase), HDAC3, histone deacetylase 3, PRMT5, protein arginine methyltransferase-5, a histone methyl transferase. Red fluorescence, AF-568 labeled antibody to specific epigenetic regulator gene; green fluorescence, α-smooth muscle actin; blue fluorescence, DAPI, DNA nuclear stain. White arrows, endothelium. Bar = 20 microns.

In order to confirm parallel changes at the protein level, we performed immunohistofluorescence analysis on paraffin-embedded sections of the proximal and distal ends of the LCCA and aortic segments studied for arterial stiffness parameters (PWV, strain measurements). We were limited to antibodies validated for fixed, paraffin-embedded section immunostaining. As shown in Figure 6, analysis of a histone acetyltransferase, Ep300 which is increased >10-fold, a histone deacetylase, HDAC3 which is increased 4.6-fold, and a histone methyltransferase, Prmt5 increased 4.9-fold, immunohistofluorescence staining detected increased protein levels in the LCCA. Interestingly, increased protein levels were detected in all vessel layers: endothelium (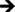), media and adventitia. As positive control, we co-immunostained for alpha-smooth muscle actin to clarify negative expression in nSP LCCA sections for all three epigenetic regulators tested: Ep300, HDAC3, and Prmt5 (Figure 6). Co-localization with DAPI staining, a DNA nuclear stain, corroborates expression of epigenetic regulators in the nucleus.

## Discussion

The experimental demonstration that arterial stiffness, measured as PWV, is increased prior to increases in SBP, DBP and PP, measured by non-invasive 24/7 telemetry, in female stroke-prone inbred Dahl Salt-sensitive (S) rats compared to contemporaneous age- and sex-matched, genetically identical non-stroke prone Dahl S rats confirms the temporal sequence of arterial stiffness and salt-sensitive essential hypertension. Given that the only difference between SP and nSP rats is the diet content of NaCl, from 0.4% NaCl in SP rats and 0.23% NaCl in nSP rats, the data provide holistic in vivo evidence on the causal role of sodium intake in the induction of arterial stiffness given salt-sensitivity. The observations in this in vivo study help unify in vitro studies on the role of sodium on endothelial cell stiffness [32], on sodium-induced endothelial glycocalyx alterations [31], and clinical studies on the impact of sodium on arterial stiffness in hypertensive patients [45].

The demonstration of causal temporal relationship is supported by the observation of molecular changes in ECM homeostasis and EC biology consistent with changes observed for fibronectin, different integrins, and collagens [45], as well as by the detection of changes implicating other genes involved in ECM structural constituents, cell adhesion proteins and regulatory matricellular proteins. While not all inclusive, the detection of pathway-specific gene changes involved in ECM and EC homeostasis and diverse functionalities demonstrates that increased sodium induces molecular changes in the vessel wall, with projected or known functional repercussions on arterial stiffness. Additionally, the observation that molecular-functional changes precede classical structural changes associated with PWV changes clarifies a pathophysiological mechanism for arterial stiffness based on molecular-functional-structural paradigms rather than a structural-functional paradigm. The apparent lag between molecular-functional changes and structure-function changes reaffirms value-added information in physiological transcriptomic analysis. We note however, that the downregulation of eNOS (NOS3) by sodium reported [35] was not confirmed here; rather we detected increased NOS3 at 6 weeks. This could suggest that NOS3 downregulation occur later.

The observation of gene-network changes collectively affecting endothelial biology, vascular ECM balance, and epigenetic regulators in all layers of the vessel wall indicates that a whole-vessel wall response occurs in increased salt intake-induced arterial stiffness. These observations are concordant with observed in vitro sodium-induced changes in the endothelium and its glycocalyx [31] and with observations of increased sodium in the vessel wall interstitium [37]. This suggests a multi-layer vessel-wall response to increased sodium involving multiple gene pathways and a complex-interacting vascular molecular paradigm at the onset of arterial stiffness. Furthermore, observed changes in epigenetic regulators spanning multiple HDAC modifiers, along with EC and ECM gene-network changes could altogether provide a self-sustaining molecular mechanism of sodium-induced vascular changes, which cumulatively increase susceptibility towards adult-onset hypertension and subsequent progression to end-organ damage. Further study of this unifying hypothesis remains to be done.

## Conclusions

In summary, this study models sodium-induced arterial stiffness which precedes hypertension in a stroke-prone salt-sensitive hypertensive rat model. Arterial stiffness detected at 6 weeks of age is associated with complex molecular changes involving ECM homeostasis, EC biology and epigenetic regulation which are detected prior to histological structural changes. Modulation of epigenetic histone modifiers indicates a mechanism for the propagation of altered steady states in the vessel wall affecting endothelial, smooth muscle and adventitial cells, thus identifying a putative molecular paradigm for the progression to hypertension, vascular disease and stroke from sodium-induced vascular changes marked by arterial stiffness.

## Materials and Methods

### Ethics statement

This study was performed in strict accordance with the recommendations in the Guide for the Care and Use of Laboratory Animals of the National Institutes of Health. The protocol was approved by the Committee on the Ethics of Animal Experiments of Boston University School of Medicine (Permit Number: AN-14966). All surgery was performed under sodium pentobarbital anesthesia, and every effort was made to minimize suffering.

### Animals

Inbred Dahl S/jrHsd rats were obtained from Harlan (Indianapolis, Indiana). Dahl S/jrHsd females×Dahl S/jrHsd males were crossed to produce progeny for subsequent studies. The stroke-prone (SP) subjects were maintained on a regular rat chow Purina 5001 containing 0.4% NaCl from gestation [44]. The non-stroke prone (nSP) rats were maintained on a Harlan rat chow containing 0.23% NaCl from gestation [44].

### Arterial Stiffness Measurements

We performed high-resolution ultrasonography measurement of pulse wave velocity (PWV) in rat abdominal aorta and left carotid artery essentially as described [43]. We used the transit time method validated for mouse carotid artery using the Vevo770 ultrasound system [43]. PWV, measured as Δd/Δt, distance (d) between two points divided by the difference in transit time (t) of the pressure wave arrival at said two points, was done reproducibly via non-invasive ultrasound micro-imaging and Doppler ultrasound transit time approach [43], based on observation in mice validating the upstroke of the velocity waveform as coincident with the pressure upstroke [46]. The transit time was defined as the time from the peak of the ECG R-wave to the foot of the velocity upstroke [43], with the foot defined as the point at the end of diastole when the steep rise of the wave front begins [47]. We used this for both the left carotid artery and abdominal aorta in 3 weeks old rats and 6 weeks old rats using optimal Vevo770 scanhead to attain optimal focal length and field of view, while maximizing high-frequency resolution and pulsed wave Doppler data acquired at 30 MHz, with 30 kHz pulse-repetition frequency (Figure 1A, 1B). The ultrasound micro-imaging and Doppler ultrasound studies were done with 0.8% isoflurane anesthesia – hence expected to not induce non-physiological artifacts since it is less than the 1% isoflurane anesthesia level defined as “baseline level” for coronary blood flow with heart rates similar to sleeping mice [48] and documented to not alter aortic impedance [49]. Measurement of strain (D^SYS^ – D^DIAS^)/D^DIAS^, where D = diameter of artery (Figure 1C), was also done in left common carotid artery as another complementary measure of arterial stiffness and distensibility. Each measurement of PWV and strain represents the average of five independent determinations per subject.

### Blood Pressure Measurements

Blood pressure (BP) was measured essentially as described [50], [51] using intra-aortic abdominal radiotelemetric implants (DATASCIENCE) obtaining non-stressed blood pressure measurements taking the average over ten-seconds every 5 minutes for 24 hours [50], [51]. Systolic (SBP), diastolic (DBP) and mean arterial pressures (MAP) were obtained along with heart rate and activity. The protocol for the SP and nSP-female rats was as follows: implant surgery at 5 weeks of age; then BP levels were collected continuously up to 16 weeks of age. The SP-rats were maintained on a Purina rat chow containing 0.4% NaCl and the nSP-rats on a Harlan diet containing 0.23% NaCl.

### Pathway-Focused Gene Expression Profiling

Gene expression profiling was done essentially as described [52]. Abdominal aorta segments (10–15 mg in weight) and left common carotid artery segments (4–6 mg in weight) were harvested from SP and nSP rats at 3 and 6 weeks of age respectively. After cold PBS perfusion under deep anesthesia, tissues were quick frozen in liquid nitrogen and stored at −80°C. RNAs from the different tissue samples were extracted with Trizol reagent (Invitrogen, CA) as described [53]. We used the rat Extracellular Matrix and Adhesion Molecules RT^2^ Profiler™ PCR Array (SABiosciences, MD) querying 84 genes related to ECM homeostasis; the rat Endothelial Cell Biology RT^2^ Profiler™ PCR Array (SABiosciences, MD) querying 84 genes related to endothelial cell biology and the rat Epigenetic Chromatin Modification Enzymes RT^2^ Profiler™ PCR Array (SABiosciences, MD) querying 84 genes representing chromatin modification enzymes known to modify genomic DNA and histones to regulate chromatin accessibility and gene expression. We performed RT-PCR analysis as per manufacturer’s instruction using 100 ng of RNA without a pre-amplification step with three biological replicates that were run in duplicates for a total of six replicates.

### Histology and Immunohistochemistry Analyses

We used the end portions of the aortic and LCCA arteries of the same arteries used for RNA isolation and characterized for PWV in order to validate correlation of gene expression changes with histological changes and PWV measurements. Artery segments for histology were fixed in buffered 4% paraformaldehyde (PFA), pH7.5, paraffin embedded and serial cross-sections were obtained. Masson Trichrome and H&E staining were done every 10-slide, and interval unstained slides were used for immunofluorescence analysis performed essentially as described [54]. All test antibodies were labeled with AF468 using the antibody labeling kit (Life technologies, Carlsbad, CA) and used for direct co-immunostaining along with alpha-smooth muscle alpha actin (aSMA) as positive control; DAPI was used to stain nuclei. Digital photomicroscopy was performed using a Zeiss Axioskop microscope. For immunofluorescence identical settings and exposure times were used in order to validate comparative analysis between SP and nSP rat artery sections. At least 4 tissue-sections per artery were analyzed for corroboration of observations.

### Statistical Analysis

All data are presented as means ± SEM. All data were analyzed for normality and descriptive statistics. The following statistical tests were performed using SigmaPlot 11.0: One Way ANOVA followed by Holm-Sidak test for multiple comparisons for analyses of arterial stiffness measurements in 3- and 6 weeks old subjects; Two Way ANOVA followed by Holm-Sidak test for multiple pairwise comparisons for blood pressure studies (diet×age) and for gene array data (gene×stroke susceptibility). A *P*<0.05 was considered statistically significant.

## Supporting Information

Table S1 **Data is presented as Ct mean ± standard deviation (three tissue samples from three independent biological replicates that were ran in duplicates, total 6 replicates); nSP, Dahl S female rats maintained in 0.23% NaCl rat diet; SP, Dahl S female rats maintained in 0.4% NaCl diet; Ct, threshold cycle; ΔCt = nSP Ct – SP Ct; Fold = 2^ΔCt^; Fold, fold increase in gene expression in SP female rats in comparison with nSP female rats;** ***P*** **, Two Way ANOVA on ranks followed by Holm-Sidak test for multiple comparisons.**
(DOCX)

Table S2 **Data is presented as Ct mean ± standard deviation (three tissue samples from three independent biological replicates that were ran in duplicates, total 6 replicates); nSP, Dahl S female rats maintained in 0.23% NaCl rat diet; SP, Dahl S female rats maintained in 0.4% NaCl diet; Ct, threshold cycle; ΔCt = nSP Ct – SP Ct; Fold = 2^ΔCt^; Fold, fold increase in gene expression in SP female rats in comparison with nSP female rats;** ***P*** **, Two Way ANOVA on ranks followed by Holm-Sidak test for multiple comparisons.**
(DOCX)

Table S3
**Data is presented as Ct mean ± standard deviation (three tissue samples from three independent biological replicates that were run in duplicates, total 6 replicates); nSP, Dahl S female rats maintained in 0.23% NaCl rat diet; SP, Dahl S female rats maintained in 0.4% NaCl diet; ND, not detected; Ct, threshold cycle; ΔCt = nSP Ct – SP Ct; Fold = 2^ΔCt^; Fold, fold increase in gene expression in SP female rats in comparison with nSP female rats;** ***P*** **, Two Way ANOVA on ranks followed by Holm-Sidak test for multiple comparisons.** 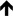, increase apoptosis; 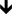, decrease apoptosis.(DOCX)

Table S4
**Data is presented as Ct mean ± standard deviation (three tissue samples from Three independent biological replicates that were ran in duplicates, total 6 replicates); nSP, Dahl S female rats maintained in 0.23% NaCl rat diet; SP, Dahl S female rats maintained in 0.4% NaCl diet; ND, not detected; Ct, threshold cycle; ΔCt = nSP Ct – SP Ct; Fold = 2^ΔCt^; Fold, fold increase in gene expression in SP female rats in comparison with nSP female rats;** ***P*** **, Two Way ANOVA on ranks followed by Holm-Sidak test for multiple comparisons.** 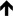, increase apoptosis; 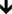, decrease apoptosis.(DOCX)

Table S5 **Data is presented as Ct mean ± standard deviation (three tissue samples from three independent biological replicates that were ran in duplicates, total 6 replicates); nSP, Dahl S female rats maintained in 0.23% NaCl rat diet; SP, Dahl S female rats maintained in 0.4% NaCl diet; Ct, threshold cycle; ΔCt = nSP Ct – SP Ct; Fold = 2^ΔCt^; Fold, fold increase in gene expression in SP female rats in comparison with nSP female rats;** ***P*** **, Two Way ANOVA on ranks followed by Holm-Sidak test for multiple comparisons.**
(DOCX)

Table S6 **Data is presented as Ct mean ± standard deviation (three tissue samples from three independent biological replicates that were ran in duplicates, total 6 replicates); nSP, Dahl S female rats maintained in 0.23% NaCl rat diet; SP, Dahl S female rats maintained in 0.4% NaCl diet; Ct, threshold cycle; ΔCt = nSP Ct – SP Ct; Fold = 2^ΔCt^; Fold, fold increase in gene expression in SP female rats in comparison with nSP female rats;** ***P*** **, Two Way ANOVA on ranks followed by Holm-Sidak test for multiple comparisons.**
(DOCX)

Table S7 **√, gene is expressed; -, gene is not expressed.**
(DOCX)

Table S8 **√, gene is expressed; -, gene is not expressed.**
(DOCX)

Table S9 **√, gene is expressed; -, gene is not expressed.**
(DOCX)
