# Supplementary material for: Aortic and Carotid Arterial Stiffness and Epigenetic Regulator Gene Expression Changes Precede Blood Pressure Rise in Stroke-Prone Dahl Salt-Sensitive Hypertensive Rats
Source: PLoS One. 2014 Sep 17;9(9):e107888. doi: 10.1371/journal.pone.0107888 (PMC4168262; doi:10.1371/journal.pone.0107888)
Supplement: Table S1 — Data is presented as Ct mean ± standard deviation (three tissue samples from three independent biological replicates that were ran in duplicates, total 6 replicates); nSP, Dahl S female rats maintained in 0.23% NaCl rat diet; SP, Dahl S female rats maintained in 0.4% NaCl diet; Ct, threshold cycle; ΔCt = nSP Ct – SP Ct; Fold = 2ΔCt; Fold, fold increase in gene expression in SP female rats in comparison with nSP female rats; P , Two Way ANOVA on ranks followed by Holm-Sidak test for multiple comparisons. (DOCX) [file pone.0107888.s001.docx]

| **Table S1. RT-PCR array profiling of extracellular matrix and adhesion molecules in left common carotid arteries from stroke-prone (SP) Dahl S female rats maintained in 0.4% NaCl rat diet compared with non stroke-prone (nSP) Dahl S female rats maintained in 0.23 % NaCl rat diet at 6 weeks of age.** | | | | | | |
| --- | --- | --- | --- | --- | --- | --- |
| ***6 weeks Left common carotid artery*** | | | | | | |
| *Transmembrane molecules* | | | | | | |
| **Gene** | **Description** | **nSP Ct** | **SP Ct** | **∆Ct** | **Fold** | ***P*** |
| *Cdh2* | Cadherin 2 | 32.58 ± 2.87 | 29.03 ± 2.27 | 3.55 | 11.70 | 0.0033 |
| *Ncam1* | Neural cell adhesion molecule 1 | 33.28 ± 2.46 | 29.30 ± 1.75 | 3.98 | 15.75 | 0.0005 |
| *Ncam2* | Neural cell adhesion molecule 2 | 38.91 ± 2.68 | 33.90 ± 4.18 | 5.01 | 32.00 | 0.0004 |
| *Syt1* | Synaptotagmin I | 40.00 ± 0.00 | 33.72 ± 5.56 | 6.28 | 77.91 | < 0.0001 |
| *Cell-cell adhesion* | | | | | | |
| *Cdh1* | Cadherin 1 | 36.38 ± 1.41 | 31.77 ± 1.50 | 4.61 | 24.48 | 0.0010 |
| *Cell-matrix adhesion* | | | | | | |
| *Itgav* | Integrin, alpha V | 30.64 ± 0.36 | 28.04 ± 0.55 | 2.60 | 6.10 | 0.0170 |
| *Itga4* | Integrin, alpha 4 | 33.96 ± 3.39 | 30.91 ± 1.81 | 3.05 | 8.26 | 0.0350 |
| *Itga2* | Integrin, alpha 2 | 38.78 ± 1.89 | 34.84 ± 4.14 | 3.94 | 15.35 | 0.0029 |
| *Itgb4* | Integrin, beta 4 | 36.60 ± 2.65 | 31.30 ± 5.08 | 5.30 | 39.37 | < 0.0001 |
| *Itga3* | Integrin, alpha 3 | 36.40 ± 1.70 | 33.56 ± 1.72 | 2.84 | 7.16 | 0.144 |
| *Ctgf* | Connective tissue growth factor | 31.20 ± 1.46 | 30.57 ± 1.38 | 0.63 | 1.55 | 0.537 |
| *Itga5* | Integrin, alpha 5 | 33.05 ± 1.57 | 31.21 ± 1.20 | 1.84 | 3.58 | 0.157 |
| *Other adhesion molecules* | | | | | | |
| *Catna1* | Catenin (cadherin associated protein), alpha 1 | 30.44 ± 0.37 | 28.03 ± 1.21 | 2.41 | 5.32 | 0.0290 |
| *Tgfbi* | Transforming growth factor, beta induced | 32.22 ± 1.18 | 29.25 ± 2.50 | 2.97 | 7.86 | 0.0070 |
| *Ctnna2* | Catenin (cadherin associated protein), alpha 2 | 40.00 ± 0.00 | 35.99 ± 3.78 | 4.01 | 16.11 | 0.0150 |
| *Vcan* | Versican | 30.12 ± 0.58 | 30.38 ± 0.73 | -0.26 | -1.20 | 0.783 |
| *Lama3* | Laminin, alpha 3 | 36.47 ± 2.46 | 33.28 ± 2.44 | 3.19 | 9.12 | 0.106 |
| *Fn1* | Fibronectin 1 | 31.64 ± 1.01 | 33.37 ± 5.58 | -1.73 | -3.32 | 0.641 |
| *Thbs2* | Thrombospondin 2 | 33.01 ± 0.60 | 34.14 ± 5.48 | -1.13 | -2.19 | 0.735 |
| *Basement membrane constituents* | | | | | | |
| *Lama2* | Laminin, alpha 2 | 28.54 ± 2.25 | 25.51 ± 0.51 | 3.03 | 8.13 | 0.0250 |
| *Collagens and ECM structural constituents* | | | | | | |
| *Col2a1* | Collagen, type II, alpha 1 | 40.00 ± 0.00 | 34.06 ± 3.60 | 5.94 | 61.50 | < 0.0001 |
| *Col8a1* | Collagen, type VIII, alpha 1 | 38.04 ± 2.26 | 35.30 ± 2.02 | 2.74 | 6.68 | 0.145 |
| *ECM proteases* | | | | | | |
| *Mmp14* | Matrix metallopeptidase 14 (membrane-inserted) | 34.89 ± 2.58 | 30.80 ± 1.92 | 4.09 | 17.03 | 0.0010 |
| *Mmp15* | Matrix metallopeptidase 15 | 36.10 ± 0.61 | 32.13 ± 2.10 | 3.97 | 15.62 | 0.0030 |
| *ECM protease inhibitors* | | | | | | |
| *Timp1* | TIMP metallopeptidase inhibitor 1 | 28.56 ± 2.32 | 26.71 ± 1.54 | 1.85 | 3.60 | 0.423 |
| *Other ECM molecules* | | | | | | |
| *Spock1* | Sparc/osteonectin (testican) 1 | 36.07 ± 0.77 | 32.52 ± 1.66 | 3.55 | 11.70 | 0.0130 |
