# Supplementary material for: Aortic and Carotid Arterial Stiffness and Epigenetic Regulator Gene Expression Changes Precede Blood Pressure Rise in Stroke-Prone Dahl Salt-Sensitive Hypertensive Rats
Source: PLoS One. 2014 Sep 17;9(9):e107888. doi: 10.1371/journal.pone.0107888 (PMC4168262; doi:10.1371/journal.pone.0107888)
Supplement: Table S2 — Data is presented as Ct mean ± standard deviation (three tissue samples from three independent biological replicates that were ran in duplicates, total 6 replicates); nSP, Dahl S female rats maintained in 0.23% NaCl rat diet; SP, Dahl S female rats maintained in 0.4% NaCl diet; Ct, threshold cycle; ΔCt = nSP Ct – SP Ct; Fold = 2ΔCt; Fold, fold increase in gene expression in SP female rats in comparison with nSP female rats; P , Two Way ANOVA on ranks followed by Holm-Sidak test for multiple comparisons. (DOCX) [file pone.0107888.s002.docx]

| **Table S2. RT-PCR array profiling of extracellular matrix and adhesion molecules in aortas from stroke-prone (SP) Dahl S female rats maintained in 0.4% NaCl rat diet compared with non stroke-prone (nSP) Dahl S female rats maintained in 0.23 % NaCl rat diet at 6 weeks of age.** | | | | | | |
| --- | --- | --- | --- | --- | --- | --- |
| ***6 weeks Aorta*** | | | | | | |
| *Transmembrane molecules* | | | | | | |
| **Gene** | **Description** | **nSP Ct** | **SP Ct** | **∆Ct** | **Fold** | ***P*** |
| *Cdh2* | Cadherin 2 | 39.25 ± 1.74 | 37.23 ± 3.14 | 2.02 | 4.05 | 0.194 |
| *Ncam1* | Neural cell adhesion molecule 1 | 32.46 ± 0.71 | 31.35 ± 0.81 | 1.11 | 2.16 | 0252 |
| *Ncam2* | Neural cell adhesion molecule 2 | 38.80 ± 1.93 | 40.00 ± 0.00 | -1.20 | -2.30 | 0.475 |
| *Syt1* | Synaptotagmin I | 40.00 ± 0.00 | 39.67 ± 0.81 | 0.33 | 1.26 | 0.956 |
| *Cell-cell adhesion* | | | | | | |
| *Cdh1* | Cadherin 1 | 36.13 ± 1.27 | 34.83 ± 1.27 | 1.30 | 2.46 | 0.55 |
| *Cell-matrix adhesion* | | | | | | |
| *Itgav* | Integrin, alpha V | 30.58 ± 0.60 | 29.71 ± 0.87 | 0.87 | 1.83 | 0.47 |
| *Itga4* | Integrin, alpha 4 | 35.64 ± 2.13 | 33.47 ± 1.19 | 2.17 | 4.50 | 0.0500 |
| *Itga2* | Integrin, alpha 2 | 38.40 ± 2.47 | 38.47 ± 2.28 | -0.07 | -1.05 | 0.995 |
| *Itgb4* | Integrin, beta 4 | 33.85 ± 1.61 | 32.30 ± 0.65 | 1.55 | 2.93 | 0.11 |
| *Itga3* | Integrin, alpha 3 | 33.78 ± 2.23 | 31.82 ± 2.21 | 1.96 | 3.89 | 0.0140 |
| *Ctgf* | Connective tissue growth factor | 32.05 ± 1.98 | 29.63 ± 0.70 | 2.42 | 5.35 | 0.0003 |
| *Itga5* | Integrin, alpha 5 | 32.10 ± 1.69 | 29.49 ± 0.76 | 2.61 | 6.11 | 0.0001 |
| *Other adhesion molecules* | | | | | | |
| *Catna1* | Catenin (cadherin associated protein), alpha 1 | 30.70 ± 1.01 | 29.56 ± 0.70 | 1.14 | 2.20 | 0.191 |
| *Tgfbi* | Transforming growth factor, beta induced | 32.00 ± 0.99 | 31.26 ± 0.80 | 0.74 | 1.67 | 0.567 |
| *Ctnna2* | Catenin (cadherin associated protein), alpha 2 | 39.99 ± 0.02 | 40.00 ± 0.00 | -0.01 | -1.01 | 0.983 |
| *Vcan* | Versican | 32.57 ± 1.44 | 30.90 ± 0.64 | 1.67 | 3.18 | 0.0291 |
| *Lama3* | Laminin, alpha 3 | 37.18 ± 2.15 | 33.18 ± 1.16 | 4.00 | 16.00 | 0.0005 |
| *Fn1* | Fibronectin 1 | 31.38 ± 1.88 | 29.49 ± 0.38 | 1.89 | 3.71 | 0.0075 |
| *Thbs2* | Thrombospondin 2 | 34.87 ± 2.61 | 32.86 ± 2.71 | 2.01 | 4.03 | 0.0088 |
| *Basement membrane constituents* | | | | | | |
| *Lama2* | Laminin, alpha 2 | 32.68 ± 0.79 | 32.60 ± 0.62 | 0.08 | 1.06 | 1 |
| *Collagens and ECM structural constituents* | | | | | | |
| *Col2a1* | Collagen, type II, alpha 1 | 40.00 ± 0.00 | 39.36 ± 1.54 | 0.64 | 1.56 | 0.885 |
| *Col8a1* | Collagen, type VIII, alpha 1 | 35.14 ± 3.00 | 31.78 ± 1.17 | 3.36 | 10.27 | 0.0002 |
| *ECM proteases* | | | | | | |
| *Mmp14* | Matrix metallopeptidase 14 (membrane-inserted) | 34.47 ± 2.15 | 32.32 ± 0.69 | 2.15 | 4.44 | 0.0272 |
| *Mmp15* | Matrix metallopeptidase 15 | 36.27 ± 1.12 | 34.73 ± 1.05 | 1.54 | 2.91 | 0.392 |
| *ECM protease inhibitors* | | | | | | |
| *Timp1* | TIMP metallopeptidase inhibitor 1 | 32.75 ± 1.91 | 30.92 ± 0.88 | 1.83 | 3.56 | 0.0190 |
| *Other ECM molecules* | | | | | | |
| *Spock1* | Sparc/osteonectin (testican) 1 | 34.77 ± 1.95 | 33.86 ± 0.97 | 0.91 | 1.88 | 0.786 |
