# Supplementary material for: Aortic and Carotid Arterial Stiffness and Epigenetic Regulator Gene Expression Changes Precede Blood Pressure Rise in Stroke-Prone Dahl Salt-Sensitive Hypertensive Rats
Source: PLoS One. 2014 Sep 17;9(9):e107888. doi: 10.1371/journal.pone.0107888 (PMC4168262; doi:10.1371/journal.pone.0107888)
Supplement: Table S3 — Data is presented as Ct mean ± standard deviation (three tissue samples from three independent biological replicates that were run in duplicates, total 6 replicates); nSP, Dahl S female rats maintained in 0.23% NaCl rat diet; SP, Dahl S female rats maintained in 0.4% NaCl diet; ND, not detected; Ct, threshold cycle; ΔCt = nSP Ct – SP Ct; Fold = 2ΔCt; Fold, fold increase in gene expression in SP female rats in comparison with nSP female rats; P , Two Way ANOVA on ranks followed by Holm-Sidak test for multiple comparisons. , increase apoptosis; , decrease apoptosis. (DOCX) [file pone.0107888.s003.docx]

| **Table S3. RT-PCR array profiling of endothelial cell function genes in left common carotid arteries from stroke-prone (SP) Dahl S female rats maintained in 0.4% NaCl rat diet compared with non stroke-prone (nSP) Dahl S female rats maintained in 0.23 % NaCl rat diet at 6 weeks of age.** | | | | | | |
| --- | --- | --- | --- | --- | --- | --- |
| ***6 weeks Left common carotid artery*** | | | | | | |
| *Angiogenesis* | | | | | | |
| **Gene** | **Description** | **nSP Ct** | **SP Ct** | **∆Ct** | **Fold** | ***P*** |
| *Adam17* | ADAM metallopeptidase domain 17 | 38.54 ± 3.56 | 34.65 ± 5.47 | 3.89 | 14.84 | 0.0030 |
| *Cdh5* | Cadherin 5 | 31.78 ± 0.28 | 29.31 ± 1.46 | 2.47 | 5.53 | 0.0140 |
| *Cxcl1* | Chemokine (C-X-C motif) ligand 1 | 29.04 ± 0.51 | 28.15 ± 2.16 | 0.89 | 1.85 | 0.621 |
| *Ednra* | Endothelin receptor type A | 30.21 ± 0.52 | 28.80 ± 2.51 | 1.41 | 2.65 | 0.204 |
| *Fgf1* | Fibroblast growth factor 1 | 29.96 ± 0.42 | 27.94 ± 1.67 | 2.02 | 4.05 | 0.204 |
| *Npr1* | Natriuretic peptide receptor A | 33.82 ± 2.04 | 32.03 ± 1.77 | 1.79 | 3.45 | 0.195 |
| *Pgf* | Placental growth factor | ND | ND |  |  |  |
| *Serpine1* | Serpin peptidase inhibitor, clade E member1 | 35.75 ± 3.22 | 33.27 ± 3.88 | 2.48 | 5.57 | 0.054 |
| *Tek* | TEK tyrosine kinase, endothelial | 39.00 ± 1.81 | 33.20 ± 3.48 | 5.80 | 55.62 | 0.0002 |
| *Tgfb1* | Transforming growth factor, beta 1 | 36.03 ± 2.75 | 32.14 ± 1.93 | 3.89 | 14.84 | 0.0120 |
| *Tymp* | Thymidine phosphorylase | 30.28 ± 0.43 | 29.27 ± 2.16 | 1.01 | 2.01 | 0.532 |
| *Xdh* | Xanthine dehydrogenase | 29.40 ± 0.44 | 30.84 ± 4.66 | -1.44 | -2.71 | 0.737 |
| *Vasoconstriction & vasodilation* | | | | | | |
| *Ace* | Angiotensin I converting enzyme 1 | 33.79 ± 1.89 | 31.18 ± 1.90 | 2.61 | 6.12 | 0.0310 |
| *Nos3* | Nitric oxide synthase 3, endothelial cell | 33.48 ± 0.79 | 29.72 ± 0.80 | 3.76 | 13.55 | 0.0002 |
| *Ptgis* | Prostaglandin I2 (prostacyclin) synthase | 32.16 ± 0.84 | 29.84 ± 1.97 | 2.32 | 5.01 | 0.0100 |
| *Apoptosis Balance* | | | | | | |
| *Bax* | 🡹 Bcl2-associated X protein | 31.65 ± 0.64 | 28.88 ± 1.15 | 2.78 | 6.87 | 0.0026 |
| *Casp3* | 🡹 Caspase 3 | 34.20 ± 1.35 | 30.89 ± 1.21 | 3.31 | 9.92 | 0.0040 |
| *Casp6* | 🡹 Caspase 6 | 40.00 ± 0 | 34.71 ± 3.21 | 5.29 | 39.10 | 0.0017 |
| *Fas* | 🡹 Fas (TNF receptor superfamily, member 6) | 34.76 ± 4.18 | 30.66 ± 1.88 | 4.10 | 17.12 | 0.0040 |
| *Tnfsf10* | 🡹 Tumor necrosis factor superfamily, member 10 | 36.45 ± 3.10 | 32.18 ± 2.75 | 4.27 | 19.33 | 0.0040 |
| *Bcl2l1* | 🡻 Bcl2-like 1 | 38.49 ± 2.93 | 34.20 ± 4.69 | 4.29 | 19.55 | 0.0015 |
| *Ippk* | 🡻 Inositol 1,3,4,5,6-pentakisphosphate 2-kinase | 37.98 ± 3.15 | 34.50 ± 4.40 | 3.48 | 11.14 | 0.0410 |
| *Rhob* | 🡻 Ras homolog gene family, member B | 31.86 ± 0.47 | 29.58 ± 2.23 | 2.28 | 4.85 | 0.0190 |
|  |  |  |  |  |  |  |
| *Coagulation/Platelet activation* | | | | | | |
| *Pdgfra* | Platelet derived growth factor receptor, alpha | 36.80 ± 3.65 | 34.74 ± 3.67 | 2.06 | 4.16 | 0.25 |
| *Plau* | Plasminogen activator, urokinase | 36.31 ± 4.14 | 33.30 ± 4.46 | 3.01 | 8.04 | 0.0450 |
| *Thbd* | Thrombomodulin | 37.10 ± 1.62 | 33.48 ± 3.49 | 3.62 | 12.32 | 0.0110 |
