# Supplementary material for: Aortic and Carotid Arterial Stiffness and Epigenetic Regulator Gene Expression Changes Precede Blood Pressure Rise in Stroke-Prone Dahl Salt-Sensitive Hypertensive Rats
Source: PLoS One. 2014 Sep 17;9(9):e107888. doi: 10.1371/journal.pone.0107888 (PMC4168262; doi:10.1371/journal.pone.0107888)
Supplement: Table S4 — Data is presented as Ct mean ± standard deviation (three tissue samples from Three independent biological replicates that were ran in duplicates, total 6 replicates); nSP, Dahl S female rats maintained in 0.23% NaCl rat diet; SP, Dahl S female rats maintained in 0.4% NaCl diet; ND, not detected; Ct, threshold cycle; ΔCt = nSP Ct – SP Ct; Fold = 2ΔCt; Fold, fold increase in gene expression in SP female rats in comparison with nSP female rats; P , Two Way ANOVA on ranks followed by Holm-Sidak test for multiple comparisons. , increase apoptosis; , decrease apoptosis. (DOCX) [file pone.0107888.s004.docx]

| **Table S4. RT-PCR array profiling of endothelial cell function genes in aortas from stroke-prone (SP) Dahl S female rats maintained in 0.4% NaCl rat diet compared with non stroke-prone (nSP) Dahl S female rats maintained in 0.23 % NaCl rat diet at 6 weeks of age.** | | | | | | |
| --- | --- | --- | --- | --- | --- | --- |
| ***6 weeks Aorta*** | | | | | | |
| *Angiogenesis* | | | | | | |
| **Gene** | **Description** | **nSP Ct** | **SP Ct** | **∆Ct** | **Fold** | ***P*** |
| *Adam17* | ADAM metallopeptidase domain 17 | 34.01 ± 3.08 | 31.82 ± 1.54 | 2.19 | 4.56 | 0.118 |
| *Cdh5* | Cadherin 5 | 29.69 ± 0.28 | 30.44 ± 1.10 | -0.75 | -1.68 | 0.453 |
| *Cxcl1* | Chemokine (C-X-C motif) ligand 1 | 30.62 ± 1.54 | 32.39 ±1.24 | -1.77 | -3.42 | 0.0023 |
| *Ednra* | Endothelin receptor type A | 30.32 ± 0.41 | 31.61 ± 1.48 | -1.29 | -2.44 | 0.0360 |
| *Fgf1* | Fibroblast growth factor 1 | 30.70 ± 0.71 | 32.47 ± 1.55 | -1.77 | -3.42 | 0.0052 |
| *Npr1* | Natriuretic peptide receptor A | 32.94 ± 0.87 | 31.16 ± 0.74 | 1.78 | 3.44 | 0.0027 |
| *Pgf* | Placental growth factor | 38.70 ± 2.92 | 33.97 ± 1.61 | 4.73 | 26.46 | 0.0001 |
| *Serpine1* | Serpin peptidase inhibitor, clade E member1 | 34.44 ± 3.33 | 30.43 ± 1.29 | 4.01 | 16.07 | <0.0001 |
| *Tek* | TEK tyrosine kinase, endothelial | 31.67 ± 0.65 | 31.38 ± 1.45 | 0.29 | 1.22 | 0.415 |
| *Tgfb1* | Transforming growth factor, beta 1 | 32.46 ± 0.37 | 32.69 ± 1.42 | -0.23 | -1.17 | 0.865 |
| *Tymp* | Thymidine phosphorylase | 31.67 ± 1.52 | 33.26 ± 1.40 | -1.59 | -3.01 | 0.0196 |
| *Xdh* | Xanthine dehydrogenase | 30.06 ± 0.43 | 31.97 ± 1.47 | -1.91 | -3.76 | 0.0008 |
| *Vasoconstriction & vasodilation* | | | | | | |
| *Ace* | Angiotensin I converting enzyme 1 | 31.64 ± 1.18 | 31.41 ± 0.96 | 0.23 | 1.17 | 0.684 |
| *Nos3* | Nitric oxide synthase 3, endothelial cell | 30.91 ± 0.45 | 31.58 ± 1.39 | -0.67 | -1.59 | 0.388 |
| *Ptgis* | Prostaglandin I2 (prostacyclin) synthase | 27.98 ± 0.22 | 27.79 ± 0.76 | 0.19 | 1.14 | 0.967 |
| *Apoptosis Balance* | | | | | | |
| *Bax* | 🡹 Bcl2-associated X protein | 30.55 ± 0.75 | 30.45 ± 0.79 | 0.1 | 1.07 | 0.81 |
| *Casp3* | 🡹 Caspase 3 | 33.19 ± 1.29 | 33.23 ± 0.80 | -0.04 | -1.03 | 0.903 |
| *Casp6* | 🡹 Caspase 6 | ND | ND |  |  |  |
| *Fas* | 🡹 Fas (TNF receptor superfamily, member 6) | 34.81 ± 2.66 | 32.93 ± 1.17 | 1.88 | 3.68 | 0.401 |
| *Tnfsf10* | 🡹 Tumor necrosis factor superfamily, member 10 | 30.45 ± 0.49 | 31.55 ± 1.06 | -1.1 | -2.14 | 0.124 |
| *Bcl2l1* | 🡻 Bcl2-like 1 | 31.89 ± 1.13 | 31.49 ± 0.87 | 0.4 | 1.32 | 0.764 |
| *Ippk* | 🡻 Inositol 1,3,4,5,6-pentakisphosphate 2-kinase | ND | ND |  |  |  |
| *Rhob* | 🡻 Ras homolog gene family, member B | 29.90 ± 0.85 | 29.13 ± 0.91 | 0.77 | 1.70 | 0.451 |
|  |  |  |  |  |  |  |
| *Coagulation/Platelet activation* | | | | | | |
| *Pdgfra* | Platelet derived growth factor receptor, alpha | 39.20 ± 0.87 | 33.20 ± 0.83 | 6.00 | 64.00 | <0.0001 |
| *Plau* | Plasminogen activator, urokinase | 37.91 ± 2.88 | 33.88 ± 0.69 | 4.03 | 16.27 | 0.0023 |
| *Thbd* | Thrombomodulin | 33.83 ± 2.28 | 31.93 ± 1.12 | 1.9 | 3.73 | 0.023 |
