# Supplementary material for: Aortic and Carotid Arterial Stiffness and Epigenetic Regulator Gene Expression Changes Precede Blood Pressure Rise in Stroke-Prone Dahl Salt-Sensitive Hypertensive Rats
Source: PLoS One. 2014 Sep 17;9(9):e107888. doi: 10.1371/journal.pone.0107888 (PMC4168262; doi:10.1371/journal.pone.0107888)
Supplement: Table S5 — Data is presented as Ct mean ± standard deviation (three tissue samples from three independent biological replicates that were ran in duplicates, total 6 replicates); nSP, Dahl S female rats maintained in 0.23% NaCl rat diet; SP, Dahl S female rats maintained in 0.4% NaCl diet; Ct, threshold cycle; ΔCt = nSP Ct – SP Ct; Fold = 2ΔCt; Fold, fold increase in gene expression in SP female rats in comparison with nSP female rats; P , Two Way ANOVA on ranks followed by Holm-Sidak test for multiple comparisons. (DOCX) [file pone.0107888.s005.docx]

| **Table S5. RT-PCR array profiling of epigenetic chromatin modification enzymes in left common carotid arteries from stroke-prone Dahl S female rats maintained in 0.4% NaCl rat diet (SP) compared with non stroke-prone Dahl S female rats maintained in 0.23 % NaCl rat diet (nSP) at 6 weeks of age.** | | | | | | | |
| --- | --- | --- | --- | --- | --- | --- | --- |
| ***6 weeks Left common carotid artery*** | | | | | | | |
| *SET domain proteins (histone methyltransferase activity)* | | | | | | | |
| **Gene** | | **Description** | **nSP Ct** | **SP Ct** | **∆Ct** | **Fold** | ***P*** |
| *Ash1l* | | Ash1 (absent, small, or homeotic)-like (Drosophila) | 32.92 ± 0.84 | 31.30 ± 4.35 | 1.62 | 3.08 | 0.0002 |
| *Setd4* | | SET domain containing 4 | 31.65 ± 1.21 | 29.52 ± 0.63 | 2.13 | 4.39 | 0.0220 |
| *DNA methyl transferases* | | | | | | | |
| *Dnmt3a* | | DNA (cytosine-5-)-methyltransferase 3 alpha | 33.33 ± 1.52 | 31.34 ± 1.81 | 1.99 | 3.97 | 0.078 |
| *Dnmt1* | | DNA (cytosine-5-)-methyltransferase 1 | 37.86 ± 3.31 | 34.03 ± 3.33 | 3.83 | 14.32 | 0.122 |
| *Histone phosphorylation* | | | | | | | |
| *Rps6ka5* | | Ribosomal protein S6 kinase, polypeptide 5 | 32.45 ± 1.00 | 29.82 ± 0.40 | 2.63 | 6.19 | 0.0050 |
| *Baz1b* | | Bromodomain adjacent to zinc finger domain, 1B | 34.31 ± 2.58 | 32.55 ± 3.20 | 1.76 | 3.39 | 0.160 |
| *Nek6* | | NIMA (never in mitosis gene a)-related kinase 6 | 37.29 ± 3.07 | 34.56 ± 5.95 | 2.73 | 6.62 | 0.0090 |
| *Histone deacetylases* | | | | | | | |
| *Sirt1* | | Sirtuin 1 | 32.35 ± 0.63 | 30.58 ± 2.23 | 1.77 | 3.39 | 0.0450 |
| *Hdac3* | | Histone deacetylase 3 | 33.18 ± 0.91 | 30.98 ± 1.70 | 2.20 | 4.61 | 0.0330 |
| *Ncor1* | | Nuclear receptor co-repressor 1 | 33.90 ± 3.28 | 30.13 ± 1.43 | 3.77 | 13.64 | 0.0010 |
| *Hdac7* | | Histone deacetylase 7 | 31.68 ± 0.90 | 30.12 ± 0.75 | 1.56 | 2.95 | 0.171 |
| *Hdac4* | | Histone deacetylase 4 | 32.63 ± 0.52 | 31.14 ± 2.06 | 1.49 | 2.81 | 0.107 |
| *Histone ubiquitination* | | | | | | | |
| *Rnf40* | | Ring finger protein 40 | 33.57 ± 0.59 | 31.16 ± 1.13 | 2.41 | 5.28 | 0.0430 |
| *Usp16* | | Ubiquitin specific peptidase 16 | 31.81 ± 1.59 | 29.36 ± 1.05 | 2.45 | 5.46 | 0.0100 |
| *Rnf2* | | Ring finger protein 2 | 31.95 ± 0.98 | 29.29 ± 0.90 | 2.66 | 6.33 | 0.0017 |
| *Ube2b* | Ubiquitin-conjugating enzyme E2B (RAD6 homolog) | | 28.93 ± 2.01 | 26.57 ± 0.29 | 2.36 | 5.13 | 0.226 |
| *Ube2a* | | Ubiquitin-conjugating enzyme E2A(RAD6 homolog) | 30.44 ± 2.14 | 27.71 ± 1.08 | 2.73 | 6.67 | 0.0160 |
| *Rnf20* | | Ring finger protein 20 | 38.23 ± 2.74 | 34.42 ± 5.05 | 3.81 | 14.01 | 0.0130 |
| *Histone acetyltransferases* | | | | | | | |
| *Crebbp* | | CREB binding protein | 32.61 ± 1.81 | 32.86 ± 5.87 | -0.25 | -1.21 | 0.196 |
| *Kat5* | | K(lysine) acetyltransferase 5 | 32.93 ± 2.86 | 31.80 ± 4.53 | 1.13 | 2.19 | 0.07 |
| *Ep300* | | E1A binding protein p300 | 36.04 ± 2.21 | 31.89 ± 3.13 | 4.15 | 17.84 | 0.0030 |
| *Hat1* | | Histone acetyltransferase 1 | 30.66 ± 2.54 | 27.83 ± 0.99 | 2.83 | 7.12 | 0.0120 |
| *Med24* | | Mediator complex subunit 24 | 30.00 ± 2.12 | 27.89 ± 1.05 | 2.11 | 4.32 | 0.127 |
| *Myst2* | | MYST histone acetyltransferase 2 | 32.81 ± 0.65 | 33.37 ± 5.15 | -0.56 | -1.47 | 0.384 |
| *Kat2a* | | K(lysine) acetyltransferase 2A | 38.16 ± 2.86 | 35.28 ± 5.17 | 2.88 | 7.36 | 0.059 |
| *Myst3* | | MYST histone acetyltransferase 3 | 34.28 ± 2.68 | 30.66 ± 3.41 | 3.62 | 12.35 | 0.0007 |
| *Myst1* | | MYST histone acetyltransferase 1 | 37.26 ± 2.29 | 31.94 ± 3.16 | 5.32 | 39.83 | 0.0008 |
| *Histone methyltransferases* | | | | | | | |
| *Prmt2* | | Protein arginine methyltransferase 2 | 30.63 ± 1.21 | 28.64 ± 0.47 | 1.99 | 3.98 | 0.0200 |
| *Prmt5* | | Protein arginine methyltransferase 5 | 36.99 ± 3.29 | 34.71 ± 5.80 | 2.28 | 4.87 | 0.0220 |
| *Mll2* | | Myeloid/lymphoid or mixed-lineage leukemia 2 | 33.86 ± 3.80 | 32.26 ± 4.78 | 1.6 | 3.03 | 0.092 |
| *Prmt1* | | Protein arginine methyltransferase 1 | 33.75 ± 3.11 | 30.92 ± 4.15 | 2.83 | 7.12 | 0.0120 |
| *Prdm2* | | PR domain containing 2, with ZNF domain | 36.24 ± 2.85 | 33.85 ± 4.86 | 2.39 | 5.22 | 0.0260 |
| *Mll1* | | Myeloid/lymphoid or mixed-lineage leukemia 1 | 32.79 ± 0.73 | 31.37 ± 2.68 | 1.42 | 2.68 | 0.118 |
| *Ash2l* | | Ash2 (absent, small, or homeotic)-like (Drosophila) | 34.36 ± 2.40 | 31.29 ± 1.43 | 3.07 | 8.40 | 0.062 |
| *Dot1l* | | DOT1-like, histone H3 methyltransferase | 36.34 ± 2.19 | 31.99 ± 1.74 | 4.35 | 20.45 | 0.0250 |
| *Ehmt2* | | Euchromatic histone lysine N-methyltransferase 2 | 33.11 ± 0.61 | 30.67 ± 0.92 | 2.44 | 5.40 | 0.0210 |
| *Smyd1* | | SET and MYND domain containing 1 | 31.60 ± 1.65 | 28.20 ± 0.31 | 3.40 | 12.35 | 0.0007 |
