# Supplementary material for: Aortic and Carotid Arterial Stiffness and Epigenetic Regulator Gene Expression Changes Precede Blood Pressure Rise in Stroke-Prone Dahl Salt-Sensitive Hypertensive Rats
Source: PLoS One. 2014 Sep 17;9(9):e107888. doi: 10.1371/journal.pone.0107888 (PMC4168262; doi:10.1371/journal.pone.0107888)
Supplement: Table S6 — Data is presented as Ct mean ± standard deviation (three tissue samples from three independent biological replicates that were ran in duplicates, total 6 replicates); nSP, Dahl S female rats maintained in 0.23% NaCl rat diet; SP, Dahl S female rats maintained in 0.4% NaCl diet; Ct, threshold cycle; ΔCt = nSP Ct – SP Ct; Fold = 2ΔCt; Fold, fold increase in gene expression in SP female rats in comparison with nSP female rats; P , Two Way ANOVA on ranks followed by Holm-Sidak test for multiple comparisons. (DOCX) [file pone.0107888.s006.docx]

| **Table S6. RT-PCR array profiling of epigenetic chromatin modification enzymes in aortas from stroke-prone Dahl S female rats maintained in 0.4% NaCl rat diet (SP) compared with non stroke-prone Dahl S female rats maintained in 0.23 % NaCl rat diet (nSP) at 6 weeks of age.** | | | | | | | |
| --- | --- | --- | --- | --- | --- | --- | --- |
| ***6 weeks Aorta*** | | | | | | | |
| *SET domain proteins (histone methyltransferase activity)* | | | | | | | |
| **Gene** | | **Description** | **nSP Ct** | **SP Ct** | **∆Ct** | **Fold** | ***P*** |
| *Ash1l* | | Ash1 (absent, small, or homeotic)-like (Drosophila) | 33.36 ± 0.77 | 32.58 ± 0.54 | 0.78 | 1.71 | 0.168 |
| *Setd4* | | SET domain containing 4 | 35.78 ± 2.29 | 36.08 ± 1.99 | -0.30 | -1.24 | 0.621 |
| *DNA methyl transferases* | | | | | | | |
| *Dnmt3a* | | DNA (cytosine-5-)-methyltransferase 3 alpha | 34.74 ± 1.54 | 32.61 ± 0.84 | 2.13 | 4.38 | 0.0001 |
| *Dnmt1* | | DNA (cytosine-5-)-methyltransferase 1 | 37.94 ± 2.20 | 34.72 ± 1.04 | 3.22 | 9.31 | 0.0210 |
| *Histone phosphorylation* | | | | | | | |
| *Rps6ka5* | | Ribosomal protein S6 kinase, polypeptide 5 | 33.36 ± 1.59 | 33.81 ± 2.00 | -0.45 | -1.37 | 0.74 |
| *Baz1b* | | Bromodomain adjacent to zinc finger domain, 1B | 34.42 ± 2.62 | 32.32 ± 0.88 | 2.10 | 4.31 | 0.0031 |
| *Nek6* | | NIMA (never in mitosis gene a)-related kinase 6 | 38.48 ± 2.48 | 34.04 ± 0.59 | 4.44 | 21.65 | 0.0005 |
| *Histone deacetylases* | | | | | | | |
| *Sirt1* | | Sirtuin 1 | 33.92 ± 1.51 | 34.15 ± 1.21 | -0.23 | -1.17 | 0.8 |
| *Hdac3* | | Histone deacetylase 3 | 33.77 ± 1.02 | 33.04 ± 0.46 | 0.73 | 1.65 | 0.245 |
| *Ncor1* | | Nuclear receptor co-repressor 1 | 32.09 ± 0.50 | 31.03 ± 0.53 | 1.06 | 2.07 | 0.064 |
| *Hdac7* | | Histone deacetylase 7 | 32.30 ± 0.49 | 31.19 ± 0.44 | 1.11 | 2.16 | 0.0280 |
| *Hdac4* | | Histone deacetylase 4 | 35.46 ± 1.83 | 33.02 ± 0.75 | 2.44 | 5.39 | 0.0006 |
| *Histone ubiquitination* | | | | | | | |
| *Rnf40* | | Ring finger protein 40 | 33.97 ± 0.74 | 33.60 ± 0.99 | 0.37 | 1.29 | 0.405 |
| *Usp16* | | Ubiquitin specific peptidase 16 | 36.59 ± 1.90 | 34.47 ± 0.88 | 2.12 | 4.32 | 0.068 |
| *Rnf2* | | Ring finger protein 2 | 33.38 ± 0.83 | 32.40 ± 1.03 | 0.98 | 1.97 | 0.052 |
| *Ube2b* | Ubiquitin-conjugating enzyme E2B (RAD6 homolog) | | 32.81 ± 0.75 | 31.73 ± 0.38 | 1.08 | 2.12 | 0.0180 |
| *Ube2a* | | Ubiquitin-conjugating enzyme E2A(RAD6 homolog) | 37.09 ± 3.38 | 34.09 ± 1.08 | 3.00 | 8.00 | 0.0330 |
| *Rnf20* | | Ring finger protein 20 | 39.05 ± 2.14 | 34.64 ± 1.03 | 4.41 | 21.20 | 0.0032 |
| *Histone acetyltransferases* | | | | | | | |
| *Crebbp* | | CREB binding protein | 32.43 ± 0.95 | 31.52 ± 0.45 | 0.91 | 1.88 | 0.0480 |
| *Kat5* | | K(lysine) acetyltransferase 5 | 33.89 ± 1.28 | 32.37 ± 1.18 | 1.52 | 2.86 | 0.0024 |
| *Ep300* | | E1A binding protein p300 | 35.45 ± 1.99 | 33.63 ± 0.47 | 1.82 | 3.54 | 0.0380 |
| *Hat1* | | Histone acetyltransferase 1 | 36.86 ± 3.46 | 34.28 ± 2.61 | 2.58 | 5.98 | 0.004 |
| *Med24* | | Mediator complex subunit 24 | 36.80 ± 3.04 | 33.54 ± 0.65 | 3.26 | 9.58 | 0.0047 |
| *Myst2* | | MYST histone acetyltransferase 2 | 36.91 ± 2.74 | 33.53 ± 0.63 | 3.38 | 10.41 | 0.0009 |
| *Kat2a* | | K(lysine) acetyltransferase 2A | 38.32 ± 2.61 | 33.98 ± 0.70 | 4.34 | 20.33 | 0.0007 |
| *Myst3* | | MYST histone acetyltransferase 3 | 33.66 ± 1.87 | 33.39 ± 0.63 | 1.27 | 2.41 | 0.337 |
| *Myst1* | | MYST histone acetyltransferase 1 | 37.58 ± 2.44 | 37.17 ± 3.08 | 0.41 | 1.33 | 0.469 |
| *Histone methyltransferases* | | | | | | | |
| *Prmt2* | | Protein arginine methyltransferase 2 | 32.03 ± 0.93 | 31.57 ± 0.92 | 0.46 | 1.38 | 0.809 |
| *Prmt5* | | Protein arginine methyltransferase 5 | 37.42 ± 2.91 | 35.70 ± 2.28 | 1.72 | 3.29 | 0.236 |
| *Mll2* | | Myeloid/lymphoid or mixed-lineage leukemia 2 | 32.71 ± 0.94 | 31.60 ± 0.51 | 1.11 | 2.16 | 0.0110 |
| *Prmt1* | | Protein arginine methyltransferase 1 | 34.31 ± 1.32 | 33.05 ± 0.69 | 1.26 | 2.39 | 0.0400 |
| *Prdm2* | | PR domain containing 2, with ZNF domain | 35.23 ± 1.15 | 33.73 ± 0.76 | 1.50 | 2.84 | 0.0490 |
| *Mll1* | | Myeloid/lymphoid or mixed-lineage leukemia 1 | 34.54 ± 1.50 | 32.61 ± 0.58 | 1.93 | 3.83 | 0.0008 |
| *Ash2l* | | Ash2 (absent, small, or homeotic)-like (Drosophila) | 37.39 ± 1.52 | 34.49 ± 0.70 | 2.90 | 7.49 | 0.0310 |
| *Dot1l* | | DOT1-like, histone H3 methyltransferase | 38.60 ± 0.98 | 34.95 ± 2.17 | 3.65 | 12.52 | 0.0044 |
| *Ehmt2* | | Euchromatic histone lysine N-methyltransferase 2 | 32.96 ± 0.38 | 32.94 ± 0.58 | 0.02 | 1.01 | 0.942 |
| *Smyd1* | | SET and MYND domain containing 1 | 32.73 ± 0.88 | 32.58 ± 0.79 | 0.15 | 1.11 | 0.973 |
