# Supplementary material for: Aortic and Carotid Arterial Stiffness and Epigenetic Regulator Gene Expression Changes Precede Blood Pressure Rise in Stroke-Prone Dahl Salt-Sensitive Hypertensive Rats
Source: PLoS One. 2014 Sep 17;9(9):e107888. doi: 10.1371/journal.pone.0107888 (PMC4168262; doi:10.1371/journal.pone.0107888)
Supplement: Table S7 — √, gene is expressed; -, gene is not expressed. (DOCX) [file pone.0107888.s007.docx]

| **Table S7**. **List of extracellular matrix and adhesion genes expressed (< 35 Ct) but unchanged.** | | | |
| --- | --- | --- | --- |
| **Gene** | **Description** | **Aorta** | **LCCA** |
| *Adamts1* | ADAM metallopeptidase with thrombospondin type 1 motif, 1 | √ | √ |
| *Adamts5* | ADAM metallopeptidase with thrombospondin type 1 motif, 5 | - | √ |
| *Col1a1* | Collagen, type I, alpha 1 | √ | √ |
| *Col3a1* | Collagen, type III, alpha 1 | √ | √ |
| *Col4a1* | Collagen, type IV, alpha 1 | √ | √ |
| *Col4a2* | Collagen, type IV, alpha 2 | √ | √ |
| *Col4a3* | Collagen, type IV, alpha 3 | √ | - |
| *Col5a1* | Collagen, type V, alpha 1 | √ | √ |
| *Col6a1* | Collagen, type VI, alpha 1 | √ | √ |
| *Ctnnb1* | Catenin (cadherin associated protein), beta 1 | √ | √ |
| *Ecm1* | Extracellular matrix protein 1 | √ | √ |
| *Entpd1* | Ectonucleoside triphosphate diphosphohydrolase 1 | √ | √ |
| *Fbln1* | Fibulin 1 | √ | √ |
| *Itgb1* | Integrin, beta 1 | √ | √ |
| *Itgb3* | Integrin, beta 3 | √ | √ |
| *Lamb2* | Laminin, beta 2 | √ | √ |
| *Lamc1* | Laminin, gamma 1 | - | √ |
| *Mmp11* | Matrix metallopeptidase 11 | √ | - |
| *Mmp1a* | Matrix metallopeptidase 1a (interstitial collagenase) | √ | √ |
| *Mmp2* | Matrix metallopeptidase 2 | √ | √ |
| *Pecam1* | Platelet/endothelial cell adhesion molecule 1 | √ | √ |
| *Postn* | Periostin, osteoblast specific factor | √ | √ |
| *Adamts2* | ADAM metallopeptidase with thrombospondin type 1 motif, 2 | √ | √ |
| *Sgce* | Sarcoglycan, epsilon | √ | √ |
| *Sparc* | Secreted protein, acidic, cysteine-rich (osteonectin) | √ | √ |
| *Spp1* | Secreted phosphoprotein 1 | √ | √ |
| *Thbs1* | Thrombospondin 1 | √ | √ |
| *Timp2* | TIMP metallopeptidase inhibitor 2 | √ | √ |
| *Timp3* | TIMP metallopeptidase inhibitor 3 | √ | √ |
