# Supplementary material for: Aortic and Carotid Arterial Stiffness and Epigenetic Regulator Gene Expression Changes Precede Blood Pressure Rise in Stroke-Prone Dahl Salt-Sensitive Hypertensive Rats
Source: PLoS One. 2014 Sep 17;9(9):e107888. doi: 10.1371/journal.pone.0107888 (PMC4168262; doi:10.1371/journal.pone.0107888)
Supplement: Table S8 — √, gene is expressed; -, gene is not expressed. (DOCX) [file pone.0107888.s008.docx]

| **Table S8**. **List of endothelial cell function genes expressed (< 35 Ct) but unchanged.** | | | |
| --- | --- | --- | --- |
| **Gene** | **Description** | **Aorta** | **LCCA** |
| *Agt* | Angiotensinogen (serpin peptidase inhibitor, clade A, member 8) | √ | √ |
| *Anxa5* | Annexin A5 | √ | √ |
| *Bcl2* | B-cell CLL/lymphoma 2 | - | √ |
| *Casp1* | Caspase 1 | - | √ |
| *Ccl2* | Chemokine (C-C motif) ligand 2 | - | √ |
| *Cflar* | CASP8 and FADD-like apoptosis regulator | √ | √ |
| *Cradd* | CASP2 and RIPK1 domain containing adaptor with death domain | √ | √ |
| *Csf2* | Colony stimulating factor 2 (granulocyte-macrophage) | - | √ |
| *Cx3cl1* | Chemokine (C-X3-C motif) ligand 1 | √ | √ |
| *Edn1* | Endothelin 1 | - | √ |
| *Edn2* | Endothelin 2 | - | √ |
| *Flt1* | Fms-related tyrosine kinase 1 | √ | - |
| *Icam1* | Intercellular adhesion molecule 1 | - | √ |
| *Il11* | Interleukin 11 | - | √ |
| *Il3* | Interleukin 3 | - | √ |
| *Kdr* | Kinase insert domain receptor | √ | √ |
| *Kit* | V-kit Hardy-Zuckerman 4 feline sarcoma viral oncogene homolog | √ | √ |
| *Nos2* | Nitric oxide synthase 2, inducible | - | √ |
| *Nppb* | Natriuretic peptide precursor B | √ | √ |
| *Pf4* | Platelet factor 4 | √ | √ |
| *Plat* | Plasminogen activator, tissue | √ | √ |
| *Sod1* | Superoxide dismutase 1, soluble | √ | √ |
| *Tfpi* | Tissue factor pathway inhibitor (lipoprotein-associated coagulation inhibitor) | √ | √ |
| *Tnf* | Tumor necrosis factor (TNF superfamily, member 2) | - | √ |
| *Vegfa* | Vascular endothelial growth factor A | √ | √ |
| *Vwf* | Von Willebrand factor | √ | - |
