# Supplementary material for: Aortic and Carotid Arterial Stiffness and Epigenetic Regulator Gene Expression Changes Precede Blood Pressure Rise in Stroke-Prone Dahl Salt-Sensitive Hypertensive Rats
Source: PLoS One. 2014 Sep 17;9(9):e107888. doi: 10.1371/journal.pone.0107888 (PMC4168262; doi:10.1371/journal.pone.0107888)
Supplement: Table S9 — √, gene is expressed; -, gene is not expressed. (DOCX) [file pone.0107888.s009.docx]

| **Table S9**. **List of epigenetic chromatin modification enzyme genes expressed (< 35 Ct) but unchanged.** | | | |
| --- | --- | --- | --- |
| **Gene** | **Description** | **Aorta** | **LCCA** |
| *Brca2* | Breast cancer 2 | - | √ |
| *Cxxc1* | CXXC finger 1 (PHD domain) | √ | √ |
| *Edf1* | Endothelial differentiation-related factor 1 | √ | √ |
| *Eed* | Embryonic ectoderm development | - | √ |
| *Ehmt1* | Euchromatic histone-lysine N-methyltransferase 1 | √ | √ |
| *Ezh2* | Enhancer of zeste homolog 2 (Drosophila) | - | √ |
| *Fbxo11* | F-box protein 11 | - | √ |
| *Hdac1* | Histone deacetylase 1 | √ | √ |
| *Hdac2* | Histone deacetylase 2 | √ | √ |
| *Hdac4* | Histone deacetylase 4 | - | √ |
| *Hdac5* | Histone deacetylase 5 | √ | - |
| *Ing3* | Inhibitor of growth family, member 3 | - | √ |
| *Mbd2* | Methyl-CpG binding domain protein 2 | √ | √ |
| *Mll5* | Myeloid/lymphoid or mixed-lineage leukemia 5 (trithorax homolog, Drosophila) | - | √ |
| *Mta2* | Metastasis associated 1 family, member 2 | √ | - |
| *Ncoa1* | Nuclear receptor coactivator 1 | √ | √ |
| *Ncoa3* | Nuclear receptor coactivator 3 | - | √ |
| *Ncoa6* | Nuclear receptor coactivator 6 | √ | √ |
| *Nsd1* | Nuclear receptor binding SET domain protein 1 | √ | √ |
| *Pak1* | P21 protein (Cdc42/Rac)-activated kinase 1 | - | √ |
| *Prmt7* | Protein arginine methyltransferase 7 | - | √ |
| *Setd5* | SET domain containing 5 | √ | √ |
| *Setd6* | SET domain containing 6 | - | √ |
| *Setdb2* | SET domain, bifurcated 2 | - | √ |
| *Sirt2* | Sirtuin (silent mating type information regulation 2 homolog) 2 (S. cerevisiae) | - | √ |
| *Suv39h1* | Defective well? -- Suppressor of variegation 3-9 homolog 1 (Drosophila) | - | √ |
| *Suv420h2* | Suppressor of variegation 4-20 homolog 2 (Drosophila) | √ | √ |
| *Sik1* | Salt-inducible kinase 1 | √ | √ |
